# Prognostic factors for the disease course and 8-year outcome in Nordic children with oligoarticular-onset juvenile idiopathic arthritis

**DOI:** 10.1186/1546-0096-12-S1-P3

**Published:** 2014-09-17

**Authors:** Suvi Peltoniemi, Ellen Nordal, Lillemor Berntson, Marite Rygg, Anders Fasth, Troels Herlin, Susan Nielsen, Marek Zak, Kristiina Aalto

**Affiliations:** 1 University of Helsinki, Helsinki, Finland; 2 Department of Pediatrics, University Hospital of North Norway, Institute of Clinical Medicine, The Arctic University of Norway, Tromsø, Norway; 3 Department of Women's and Children's Health, Uppsala University, Uppsala, Sweden; 4 Department of Pediatrics, St. Olavs Hospital, Trondheim, Norway; 5 Department of Laboratory Medicine,Children’s and Women’s Health, Norwegian University of Science and Technology, Trondheim, Norway; 6 Department of Pediatrics, University of Gothenburg, Gothenburg, Sweden; 7 Århus University Hospital, Skejby; 8 University Clinic of Pediatrics II, Rigshospitalet, Copenhagen, Denmark; 9 Department of Pediatrics, Hospital of Children and Adolescents, Helsinki, Finland

## Introduction

Juvenile idiopathic arthritis (JIA) refers to chronic childhood arthritides of unknown aetiology with onset before the age of 16 and persisting for more than 6 weeks. According to ILAR criteria patients are divided into 7 categories of which oligoarthritis is the most common one in western countries. It is further distinguished into persistent and extended oligoarthritis depending on whether the disease is confined to four or fewer joints or it extends to more than four joints after the first 6 months of the illness. Earlier studies have proposed factors that could predict the severity in oligoarticular-onset JIA such as high initial erythrocyte sedimentation rate (ESR) and involvement of upper limb. However, more studies are needed for better and earlier identification of high-risk patients to prevent possible permanent damages.

## Objectives

The aim of the study was to find out prognostic factors that would predict disease course i.e. who of oligoarticular JIA patients will have a persistent disease course and who will develop the extended form. Another target was to determine whether the main features vary between the two oligoarticular categories and to see if the outcome is different between the two groups at 8 years after disease onset.

## Methods

This study is a multicentre population-based follow-up study in the Nordic countries based on consecutive patients with a new diagnosis of JIA according to ILAR criteria. They were enrolled between 1997 and 2000 from 14 geographically defined areas in Finland, Sweden, Norway and Denmark. Information regarding clinical data, serology, and disease activity was registered at clinical visits for 8 years.

## Results

212 of the 440 JIA patients included in the 8-year study had oligoarthritis (48.2%). 134 of them had persistent and 78 extended form. Females constituted 65.7 and 76.9% of the groups, respectively. Mean age at onset was 5.7 for persistent and 5.3 for extended oligoarthritis. During the 8 years’ follow-up period 18.7% of patients with persistent and 20.5% with extended oligoarthritis developed uveitis. The percentage of positive antinuclear antibodies (ANA) was 42.5 in the persistent and 48.7 in the extended group. At 8 years the percentage of patients with active disease was 29.1% for persistent and 60.3% for extended oligoarthritis. When analyzing the data, both small joint arthritis and inflammation of joints in upper extremities independently correlated strongly to a higher number of cumulative joints (p<0.001). Neither gender, ANA positivity, age at onset, family history of rheumatic diseases, nor high ESR in the initial period of the illness correlated to the number of affected joints in a statistically significant way.

## Conclusion

The most striking finding was the poor prognosis of the extended oligoarthritis category compared to the persistent one; 60% did not reach remission vs. 29%. Upper limb involvement was a predictor of disease severity, which is in agreement with previous studies. Furthermore, arthritis of small joints predicted development to extended oligoarthritis with a high likelihood.

## Disclosure of interest

None declared.

